# Pathophysiological Responses to Conotoxin Modulation of Voltage-Gated Ion Currents

**DOI:** 10.3390/md20050282

**Published:** 2022-04-23

**Authors:** Elisabetta Tosti, Raffaele Boni, Alessandra Gallo

**Affiliations:** 1 Department of Biology and Evolution of Marine Organisms, Stazione Zoologica Anton Dohrn, 80121 Naples, Italy; tosti@szn.it (E.T.); raffaele.boni@unibas.it (R.B.); 2 Department of Sciences, University of Basilicata, 85100 Potenza, Italy

**Keywords:** conotoxins, voltage-gated ion currents, sodium, potassium, calcium, drug discovery

## Abstract

Voltage-gated ion channels are plasma membrane proteins that generate electrical signals following a change in the membrane voltage. Since they are involved in several physiological processes, their dysfunction may be responsible for a series of diseases and pain states particularly related to neuronal and muscular systems. It is well established for decades that bioactive peptides isolated from venoms of marine mollusks belonging to the Conus genus, collectively known as conotoxins, can target different types and isoforms of these channels exerting therapeutic effects and pain relief. For this reason, conotoxins are widely used for either therapeutic purposes or studies on ion channel mechanisms of action disclosure. In addition their positive property, however, conotoxins may generate pathological states through similar ion channel modulation. In this narrative review, we provide pieces of evidence on the pathophysiological impacts that different members of conotoxin families exert by targeting the three most important voltage-gated channels, such as sodium, calcium, and potassium, involved in cellular processes.

## 1. Introduction

Marine organisms produce a great variety of toxins. Among them, venomous mollusks cone snails have provided so far more than 6000 different toxins that have been isolated and characterized by more than 100 different species. Cone snails have been the object of huge interest and study since ancient times due to their shell features but mostly for their poison-associated toxins. The main physiological roles of these toxins are for self-defense from predators but also to predate themselves and to compete with other marine species, also thanks to a poisonous sting that may be fatal for humans. The venomous properties of the toxins have been revealed to exert pharmacological bioactivities, especially on a vast variety of pain-associated neurological disorders. Hence, the toxin’s level of poisonousness has been used as an efficient and beneficial pharmacological and therapeutic tool, making conids good candidates for new drug design and development [1,2,3]. Conus-derived toxins, known as conotoxins (CnTX), are venomous small peptides consisting of 5–50 amino acid residues with multiple disulfide bonds among cysteines. An old classification divided CnTX into cysteine-rich and cysteine-poor groups. At present, instead, three CnTX groups are classified based on the cysteine framework gene superfamily and the pharmacological family. The cysteine framework group is underlined by the specific organization of cysteine residues in the toxin primary structure whereas the gene superfamily is based upon the evolutionary relationship between different conopeptides. In particular, CnTXs are translated from mRNA as precursor peptides and cDNA sequencing allows the increased discoveries of new toxin sequences.

**Figure 1:**
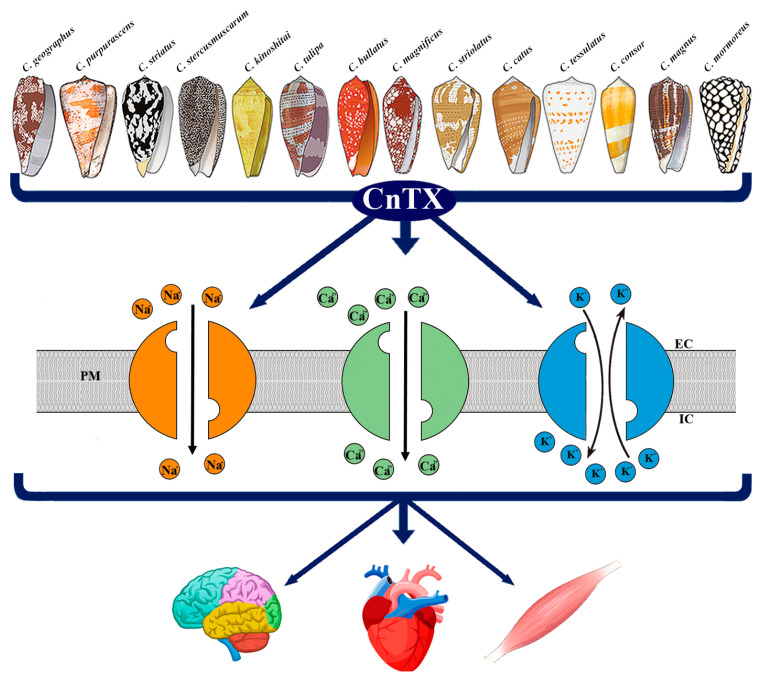
Representative image of CnTX bioactivity via voltage-gated ion channel modulation. Conus derived toxins (CnTX) target numerous and different Na_V_ and/or Ca_V_ and/or K_V_ channel subtypes generating ion current fluxes through neurons of central and peripheral nervous systems as well as in heart and skeletal muscle cells. IC = intracellular compartment; EC = extracellular compartment; PM = plasma membrane.

Currently, pharmacological family classification is related to the receptor target and the type of CnTX-target interaction. CnTX included in the same superfamily share a similar signal peptide sequence, which undergoes structural and functional differentiation when they become encoded mature peptides [4,5]. Nonetheless, over 10,000 CnTX sequences have been disclosed and published; however, 3D structural and functional information is still lacking and their pharmacological characterizations have not been elucidated. CnTX includes several different pharmacological families that selectively target specific voltage-gated ion channels, G protein-coupled receptors, enzymes, and transporters [6,7,8]. Currently, CnTXs are under evaluation by neuroscientists and drug developers for their peculiar selectivity to mammalian and human targets and, in particular, for their ability to inhibit voltage-gated ion channels. As an example, the µ-CnTX family exerts its activity by combining multiple peptides action known as “cabal”, which is aimed to ensure the most effective bioactivity through ion channels modulation. Due to their ability to interact with human ion channels, these toxins are considered “specialists in neuropharmacology” and given their therapeutic potential, some of them have been involved in human clinical treatments [9,10,11]. Based on their specific selectivity, CnTX represent basic tools to elucidate ion channel function and their involvement in biological mechanisms and processes. In this review, we focus on the main CnTX pharmacological properties and their modulation of voltage-gated sodium (Na_V_), calcium (Ca_V_), and potassium (K_V_) channels acting through different and, sometimes, opposite physiological and pathological mechanisms (Figure 1).

## 2. Ion Channels

On the plasma membrane, the different distribution of ions and related electrical charges inside and outside the cell generates an electrical gradient known as cell voltage. This trans-membrane potential named resting potential (RP) is specific in each cell type and ranges from −10 to −100 millivolts representing a store of energy for which its variation underlies a series of biological processes. In a steady-state situation, the cell membrane does not allow the free diffusion of ions that may occur only through ion channels. These are complex pore-forming proteins embedded in the plasma membrane characterized by specificity, gating, and conductance. These properties define for each ion channel which specific ion may cross it in the case of it being modulated by a change in voltage, known as voltage-gated channel, or by a ligand as well as the number of ions that may cross the channel [12]. Nonetheless, several different ions participate in the voltage generation; those mainly involved in biological mechanisms are extracellular, such as sodium (Na^+^) and calcium (Ca^2+^), and intracellular, such as potassium (K^+^).

These three cations passing through voltage-gated channels play physiological roles in generating, shaping, and transducing electrical signals in the cells. In particular, potassium (K_V_) is essential for determining and regulating the RP and cell volume, and sodium (Na_V_) is responsible for the action potential generation and propagation, whereas calcium (Ca_V_) is mainly involved in cell signaling waves and cascades [13,14,15]. From a structural point of view, voltage-gated ion channels are formed by the main pore-forming domain, which consists of either single or multiple distinct subunits. Once activated, voltage-gated ion channels undergo a conformational modification allowing ion passage through the pore; this is then deactivated/inactivated, returning quickly to a non-conducting state [16]. The ion passage through an ion channel is called ion current and is associated with a change of RP due to the transient change of ion concentrations inside the cell. The membrane depolarization is due to the shift of RP toward positive values and is associated with sodium and calcium entry, whereas hyperpolarization due to potassium exit leads RP toward negative values. A schematic pathway that links ion channel activity and biological processes arise on a sensory domain that receives physical or chemical stimuli and converts them into ion fluxes, which in turn induces a series of signal transduction cascades. A broad volume of studies reports the involvement of ion currents in a variety of physiological processes such as reproduction, gamete maturation, cell volume regulation, cell division, cardiac functionality, skeletal muscle contraction, and neuronal excitability [17,18,19,20,21,22,23,24,25,26,27,28]. Ion current activities are also involved in dysfunctions in some of the overmentioned processes. The disorders, collectively known as channelopathies, are genetic diseases resulting from the malfunction of ion channels that induce a vast array of diseases of the nervous, cardiac, respiratory, urinary endocrine, and immune systems. Among them, the best-described pathologies are hypertension, epilepsy, diabetes, blindness, cardiomyopathy, asthma, gastrointestinal disturbs, and even cancer [29]. A pivotal role in most of the known neurological channelopathies is played by Na_V_ channels dysfunction due to the key role of this channel in the generation and propagation of the action potential, the distinctive feature of neuronal activity. In fact, mutations of the three Na_V_ channels isoforms 1.1-, 1.2-, and 1.6-encoding genes have been shown to be responsible for several intellectual, behavioural, and clinical disabilities. More specifically, Na_V_ channel mutations and disorders in the conducting properties and regulation have been observed in some cases of demyelination, ischemia, and Angelman Syndrome [30,31]. The defects in ion channel functionality are caused by either genetic or acquired factors. Ion channel gene mutations are the most common cause of channelopathies, whereas drugs and toxins targeting ion channels appear to exert contrasting impacts by either impairing ion channel function and/or acting as therapeutic tools able to relieve or even treat human channelopathies [32].

## 3. Pathophysiological Response to CnTX Voltage-Gated Channel Modulation

### 3.1. Na_V_ Channels

Na_V_ channels are voltage-gated ion channels responsible for the generation of the rapid RP depolarization known as action potential that, in excitable cells, propagates electrical signals in muscles and nerves in either the central or peripheral nervous system [33]. Since Na_V_ channels underlie neurotransmission, contraction, excitation coupling, and associated physiological functions [33], mutations and defects in their functional activity are associated with several neurological disturbances and channelopathies [34]. From a structural point of view, Na_V_ channels are heteromeric complexes with a pore-forming α subunit of about 260 KDa linked to one or two β subunits of different molecular weights. The α subunit contains the binding site for several neurotoxins and drugs that target the channel and significantly change its activity. The β subunits are involved in different signaling roles in physiological processes such as cell adhesion, gene regulation, and brain development and in the kinetic regulation of channel opening. Both α and β subunits contain the receptors for toxins targeting the channel. Currently, characterized according to the α-pore-forming subunit sequences, nine isoforms of the Na_V_ channels α subunits (1.1–1.9) have been identified. Specifically, isoforms 1.1, 1.2, 1.3, and 1.6 are predominantly expressed in the central nervous system, whereas isoforms 1.7, 1.8, and 1.9 are mainly expressed in the peripheral nervous system. In addition, skeletal and heart muscles contain 1.4 and the 1.5 isoforms, respectively [35,36,37,38].

Toxins and venom compounds targeting Na_V_ channels are of particular importance due to their pivotal role played in the neuromuscular system. Together with their pathophysiological action, CnTX have also provided basic information on the molecular structure, function, and subtype-selectivity of Na_V_ channels [39] (Table 1).

Several CnTX families and, in particular, µ-, µO-, δ-, and ι′-CnTX can target Na_V_ channels upon binding specific sites on the α subunits. In particular, they may act as pore blockers that structurally occlude the pore or interfere with voltage sensors [53,54]. These families, in fact, differently modulate channel activity acting as either inhibitors (µ- and µO-CnTX) or stimulators (δ- and ι′-CnTX) of Na_V_ activity. The channel inhibition is underlined by different mechanisms. For example, µ-CnTX blocks ion conductance by binding to the channel’s external vestibule, whereas µO-CnTXs are gating modulators that bind the external side of the pore, causing channel closure [55,56]. Similarly, δ- and ι′-CnTX activate channels by two different mechanisms that prolong the channel’s opening and shift voltage activation to more hyperpolarized potentials, respectively [57]. µ-CnTX are small peptides composed of 16–22 amino acids and three disulfide bonds that, by inhibiting the α-subunit of Na_V_ channels, affect neuromuscular transmission causing paralytic to analgesic effects in mammals [58,59]. Currently, among the 12 CnTX known, μGIIIA was the first one to be isolated from *Conus geographus*; by binding the Na_V_1.4 sub-type channel pore, it exerts the inhibition of rat skeletal muscle channels [40]. From the same venom, the two isoforms μ-GIIIB and μ-GIIIC, differing for only four residues, exhibited a different affinity for the muscle subtype Na_V_ channels 1.4 and the neuronal Na_V_1.1, Na_V_1.2, and Na_V_1.6 channel subtypes. These μCnTX can discriminate between muscle and neuronal Na_V_ channels, becoming candidates for therapeutic plans on neurological disorders. Therefore, a wide range of studies was aimed to identify whether interactions between several μ-CnTX and Na_V_ channel subtypes may act as selective inhibitors of Na_V_ channels neuronal subtypes, with possible clinical impact on neurological processes [41]. In this light, several μ-CnTXs with affinities for neuronal Na_V_ channel subtypes were isolated from *Conus bullatus*, *catus*, *consor*, *kinoshitai*, *magnus*, *purpurascens*, *stercusmuscarum*, *striatus*, *striolatus*, and *tulipa*. Among these, μ-CnIIIA, μ-CnIIIB, μ-CnIIIC, μ-CIIIA, and μ-MIIIA can block the conductance of Na_V_1 subtypes in amphibian neurons. Some of them, by inhibiting olfactory and sciatic nerve action potentials, modulate pain signals and exert analgesic activity [42]; however, only CIIIA may cause paralysis [43]. μ-PIIIA is a peculiar versatile μ-CnTX that can differentiate Na_V_ channel subtype isoforms by inhibiting Na_V_1.2, Na_V_1.4, and Na_V_1.7 channel subtypes from rat brain, skeletal muscle, and peripheral nerves, respectively. Furthermore, recent studies revealed that μ-PIIIA is a strong inhibitor of Na_V_ channels involved in muscle contraction and, specifically, of Na_V_ neuronal subtypes in both the central and peripheral nervous systems [44,60]. Isolated from the venom of *Conus stercusmuscarum*, μ-SmIIIA, due to its strong affinity for neuronal Na_V_ channel subtypes, it can irreversibly block Na_V_ currents in different neurons of either amphibians or rats, demonstrating a nociceptive role [45,46]. On the contrary, a robust analgesic action is exerted in mammals by μ-SIIIA from *Conus striatus* through the neuronal block of Na_V_1.2 subtype [47]. Strong and mild affinities for neuronal Na_V_ channels were described in several μ-CnTXs, such as μ-TIIIA, μ-KIIIA, μ-KIIIB, and μ-SIIIB, which were isolated from different conids and associated with neuronal pain and analgesic activity in mice affected by inflammatory diseases [48,49,50]. In support of the role of the amino acid sequence, a recently patented invention led to the development of a µ-CnTX peptide with a specific sequence forming a bioactive fragment suitable for pharmaceutical use for either the treatment of Na_V_ channel-associated diseases or replacing anesthesia in a patient undergoing surgery [61].

In this line, μO-CnTX MrVIA, MrVIB, and MfVIA that are isolated from *Conus marmoreus* play analgesic actions on pathophysiological pains in a large variety of animal models through the inhibition of Na_V_1.8 channel activity [51]. Although δ- and ι′-CnTX stimulate Na_V_ activity, based on the induction of repetitive action potential oscillations in several cells and tissues, no clear pathophysiological evidence has been observed. Nonetheless, an electrophysiological study aimed to identify the cytotoxicity of CnTX ί-RXIA isolated from *Conus radiatus* demonstrated that this CnTX induces repetitive action potentials in frog axons and mouse sciatic nerve, possibly through the shift of voltage dependence activation of Na_V_1.6 subunits toward hyperpolarized levels. It was, therefore, assumed to be applicable in clinical applications for new neurological therapies [10,52]. Currently, increasing structural studies of Na_V_ channels have been performed in complex with toxin and drug modulators targeting either pore and/or voltage sensors. These are providing new hints on pharmacological mechanisms and can discover new isoforms of Na_V_ channels with potential applications in the future [62].

### 3.2. Ca_V_ Channels

Ca^2+^ is the signaling ion for which its elevation from the resting state is involved in many physiologic processes in most cell types. Ca^2+^ homeostasis is regulated by an intricate connection of intracellular organelles, binding molecules, and transmembrane channels and transporters. Rapid Ca^2+^ entry into the neurons gives rise to action potential propagation along cells, activating a cascade of processes such as enzyme activation and gene regulation. Ca_V_ channels are at the origin of depolarization evoked by Ca^2+^ entry into excitable cells of either brain or muscle tissues. This, in turn, is responsible for most physiological functions as Ca^2+^ dependent muscle contraction, neurotransmitters release, gene transcription, and others. Dysfunctions in these processes, therefore, may alter neurotransmission and gene transcription generating neuropathic pain and related disease states [63,64]. Ca^2+^ channels are made by 4–5 different subunits, among which the α1 includes the voltage sensor, related apparatus, and the conduction pore [65]. According to the voltage changes and depolarization amplitude needed for their activation, Ca_V_ channels are organized into two categories: high- and low-voltage activated Ca^2+^ channels. The most known members of Ca_V_ channels are (i) the high voltage-activated L-type characterized by slow voltage-dependent inactivation involved in cell excitability, contraction, gene expression regulation, and oocyte maturation; and (ii) the P/Q-, N-, and R-types that are more prominently active in fast neuronal signal transmissions [23,66]. T-types are the low voltage-activated channels present in either neurons or smooth and cardiac muscular tissues. N-type Ca_V_2.1 and Ca_V_2.2, in particular, play important roles in the transmission of pain signals to the central nervous system. About ten different genes encode for different types of Ca_V_ subunits that are grouped into three major classes (Ca_V_1, Ca_V_2, and Ca_V_3). Specifically, the Ca_V_1 family encodes four different types of L-type channels, Ca_V_2 family for 2.1, 2.2, and 2.3 corresponding to P/Q type, N-type, and R-type channels, respectively, whereas the Ca_V_3 family includes three different types of T-type Ca_V_ channels [67]. Following channel gating, once Ca^2+^ ions are released into the cytosol, they behave as second messengers binding a large number of proteins and, in turn, influence multiple cell functions and complete several physiological processes [68]. Although most of the studies have focused on the pivotal involvement of Ca_V_1.2 and Ca_V_2.2 isoforms in the modulation of pain states, some hints also revealed the involvement of T-type Ca_V_3.1–Ca_V_3.3 with a special focus on the Ca_V_3.2 knockdown effect in mechanical, thermal, and chemical pain diseases [69] (Table 2).

In the 1990s, numerous studies aimed at identifying new conopeptides, which which its homologues would exhibit possible affinity for Ca_V_ channels. However, it was only later that these peptides were characterized and their possible similarities disclosed. The in vitro studies of toxin administration and, in particular, their effects on neuropathic disturbs have been progressively evidenced, and the clinical applications for relieving different pathologies were established. The most known CnTX able to modulate Ca_V_ channels, by occluding the channel pore and, thus, preventing Ca^2+^ entry, is the ω-CnTX family. Typically, ω-CnTX are peptides that are composed of 24–30 amino acids and belong to the superfamily of disulfide-rich conopeptides [88,89]. In the ω-CnTX family, numerous peptides have been isolated from different conid venoms [90]. Depending on the molecular structure, ω-CnTXs that target neuronal N-type Ca_V_ channels have been identified as potential drugs for chronic pain treatments. The antagonism with the N-type Ca_V_ also suggested a ω-CnTX-neuroprotective effect through a size reduction in cerebral infarction and the delayed inhibition of neuronal cell death in the hippocampal CA1 area [91].

PnVIA and PnVIB from *Conus pennaceus* were among the first ω-CnTx identified to be able to discriminate subtypes of high voltage-activated (HVA) Ca^2+^ currents in molluscan neurons. In the snail *Lymnaea stagnalis*, they selectively but reversibly blocked transient HVA currents in caudodorsal cells with negligible effects on L-type currents. Although no clear effects on neurological dysfunctions were reported, they were considered useful selective drugs for relevant Ca_V_ channel subtypes [70]. Similarly, ω-CnTx TxVII from *Conus textile* showed a Ca^2+^ current block activity for which its effects were studied in the caudodorsal neurons in the mollusk *Lymnaea stagnalis*. Although preliminary studies on this CnTx did not reveal any pathophysiological effects, it was considered a promising tool for designing selective peptide probes for L-type Ca^2+^ channels [71].

GVIA from *Conus geographus* was first investigated in in vitro studies and was shown to induce an irreversible block of Ca_V_ channels in frog skeletal neuromuscular junction by preventing acetylcholine release. Furthermore, GVIA, demonstrating a role in the attenuation of the Ca^2+^ component of the action potential in the dorsal root ganglion of a chick embryo, provided useful information on presynaptic connection mechanisms [72]. MVIIA from *Conus magnus* is the most popular CnTX since, following its isolation and characterization, it gave rise to a widely used pharmacological preparation able to relieve pain in humans [92]. Ziconotide, or Prialt as a commercial name, is used in the treatment of chronic pain related to cancer pathologies; however, it also decreases spontaneous tremors and locomotor activities. By inhibiting the Ca_V_2.2 channel, MVIIA acts as an analgesic drug for which its therapeutic mechanism relies on amino acid residues such as arginine13 and tyrosine13 positions [73]. Furthermore, the different affinity for the Ca_V_2.2 channel allows discriminating MVIIA from two newly discovered ω-CnTX, MoVIA, and MoVIB, which were identified from *Conus moncuri* [75]. The search for alternative CnTX is of topical importance since ω-MVIIA induces warring side effects that severely limit its use as a pain reliever [77]. Similarly, some features of ω-MVIIA are in common with ω-MVIIC from *Conus magnus*, a peptide blocker of P/Q-type Ca_V_2.1 and Ca_V_2.2 channels that is essential in the process of neurotransmitter release underlying the development of spinal cord injury. The block of these Ca_V_ channels by the CnTX exerts a specific neuroprotective effect in rats [74]. Three paralytics ω-CnTX that were isolated and characterized in the 90 s from *Conus striatus* are SVIA, SVIB, and SO-3. SVIA targets Ca_V_2.2 channels and exerts strong paralytic effects in lower vertebrates and milder effects in mammals. SVIB, through both Ca_V_2.1 and Ca_V_2.2 channel activation, is lethal to mice when injected; however, it showed different binding affinity sites with respect to those of GVIA and/or MVIIA [76]. SO-3 is an inhibitor of the N-type Ca_V_2.2 channel and possesses structure and analgesic activity similar to MVIIA. Mainly, it attenuates either acute or chronic pain in rodents and exhibits less evident disturbing side effects than MVIIA (ziconotide) [77].

The ω-CnTX CVIE, CVIF, and CVID from *Conus catus* are neurophysiological tools useful for the potential therapeutic inhibition of nociceptive pain pathways. The first two CnTX showed a higher affinity for Ca_V_ channels in the inactivated state acting on the rat partial sciatic nerve ligation model of neuropathic pain and also significantly reduced allodynic behavior [78]. CVID is an N-type Ca_V_ channel selective CnTX displaying higher selectivity and selective antagonist activity for N-type Ca_V_2.2 with respect to P/Q-type channels. For this reason, it has been investigated and tested as an analgesic drug in clinical trials, demonstrating a beneficial action on chronic neuropathic pain with a specific ability to reduce allodynic behavior in mice [79,80].

Isolated from *Conus fulmen*, FVIA ω-CnTX displayed a remarkable effect on pain and blood pressure. By inhibiting N-type Ca_V_ 2.2, FVIA exhibited similar properties to that of MVIIA by reducing, in a dose-dependent manner, nociceptive behavior in neuropathic pain models and mechanical and thermal allodynia in the rat model. Moreover, for this CnTX, the reversible action has been considered a great advantage over MVIIA due to its potency and lowered side effects [81]. Identified from *Conus textile*, the peculiar CNVIIA ω-CnTX demonstrated an inhibitory effect on N-type Ca_V_2.2 channel accompanied by an unexpected blocking activity on the neuromuscular junction in amphibians. On the contrary, direct intracerebroventricular injection in mice was reported to cause mild tremors and shaking movements whereas higher doses of intramuscular administration in fishes resulted in paralysis and even death. Due to these peculiar characteristics, CNVIIA was suggested to represent a new selective tool for the N-type Ca_V_ channel by means of a new and unique pharmacological profile [82]. Together with the large amount of ω -CnTx, some α-CnTXs were shown to act on N-type Ca_V_ channels exerting analgesic effects. In particular, the synthesized α-CnTX PeIA showed a potent blocking activity on N-type Ca_V_ channels coupled to GABAB receptors. Potential structure–activity applications of PeIA CnTX have been suggested as an analgesic compound for drug design aimed at pain treatment [83]. More recently, the α-CnTXs Vc1.1, RgIA, and some variants, such as AuIB and MII, have been shown to possess analgesic properties through the inhibition of nicotinic acetylcholine and GABAB receptors coupled to N-type Ca_V_2.2. indicating a novel mechanism for reducing DGD neuron excitability [87]. Interestingly, the GABAB receptor expression was essential for inhibiting N-type Ca_V_2.2 channels and the development of related α-CnTX analgesia. Although this mechanism is still to be clarified, it has been experimentally shown that α-CnTX successfully acted on partial/chronic sciatic nerve ligation injury models and on allodynia relief [84,85,86].

### 3.3. K_V_ Channels

K_V_ channels are plasma membrane proteins allowing the selective outside/inside flux of K^+^ ions in response to the membrane depolarization. K_V_ current activity plays a crucial role in many biological processes and functions such as RP and cell volume regulation, propagation of action potential in nerves, cardiac and skeletal muscles, cell proliferation, differentiation, and apoptosis [93]. Furthermore, K_V_ s are essential in the regulation of Ca^2+^ six transmembrane helices (S1–S6) embedded in the lipid bilayer, forming the voltage sensor domain (S1 to S4) and the pore-forming domain (S5, S6). The voltage sensor domain induces a channel conformational change by sensing RP changes, whereas the interaction with the pore generates K^+^ ion current fluxes [94]. K_V_ channels include 12 different channel families among which a pivotal role is played by the K_V_1 family, which contains up to eight isoforms (K_V_1.1–K_V_1.8). Among them, K_V_1.3 was first detected in T-cells and, hence, is considered as a possible target for treating autoimmune diseases such as multiple sclerosis, rheumatoid arthritis, and psoriasis. Subsequently, K_V_1.3 has been proved to be widely distributed in organs and tissues and mainly expressed in both nervous and immune systems participating in several signaling pathways of either normal and/or cancer cells. In particular, K_V_1.3 channel expressions and/or alterations are involved in numerous pathophysiological processes, such as insulin and apoptosis sensitivity, neoplastic malignancy, inflammatory diseases, cognitive alterations, and anxiety [95]. More recently, it has been shown that the inhibitors of the K_V_1.3 channel reduce neuroinflammation in rodents together with Alzheimer’s and Parkinson’s disease and trauma derived brain injury likely by enabling microglia to resist depolarization stimuli [96]. Being involved in overmentioned pathologies, the K_V_1.3 channel and its blockers have been considered as safe pharmacological tools for chronic inflammatory disease therapies such as type II diabetes mellitus, obesity, and cancer [97]. Among the CnTX studied so far, a few can modulate K_V_ channels (Table 3).

In 1998, two novel peptides from the venoms of *Conus striatus* and *Conus purpurascens* were purified and characterized, demonstrating their ability to bind and block K_V_ channels. kA-CnTX SIVA caused peculiar spastic paralytic symptoms in fish and repetitive action potential oscillations in amphibian nerve-muscle tissues in response to exposure and injection [98]. The latter κ-CnTX PVIIA, belonging to a different family, was first described to bind and block K^+^ channels [99]. This peptide possesses a disulfide bridge pattern similar to those of ω- and δ-CnTX [108]. Later, it was shown that κ-PVIIA is a functional peptide able to inhibit the shaker K_V_ channel. In particular, a selectivity on the K_V_1.3 subtype was evidenced, suggesting its potential use as therapeutics for multiple sclerosis since T-cells that express a large number of K_V_1.3 channels are important mediators of autoimmunity.

In addition, K_V_1.3 blockers can ameliorate several harmful diseases, such as rheumatoid arthritis, diabetes, and dermatitis in animal models with a safety profile in rodents and primates [100]. Accurate molecular simulation techniques aimed to disclose the interaction between PVIIA and shaker K_V_ channels demonstrated the existence of two clusters of amino acids that are critical for the binding between the toxin and the ion channel. The consistency of this binding model and the experimental data indicate that the interaction between PVIIA-K_V_ and shaker K_V_ channels may be useful for the development of new therapeutic agents [109].

kM-CnTX RIIIK is a 24 amino acid peptide isolated from *Conus radiatus* venom identified as the first CnTX able to block human K_V_1.2 channels. Although structurally similar to µ-CnTX GIIIA, RIIIK inhibits shaker K_V_ expressed in Xenopus oocytes, whereas it showed no affinity with the mammalian K_V_1.1, K_V_1.3, and K_V_1.4 subtypes [101]. When administered before reperfusion, RIIIK significantly reduced the in vivo infarct size in rat hearts demonstrating a potential cardioprotective action. On the contrary, another K-CnTX RIIIJ from the same conid venom did not exert any clear cardio-protective effects when targeting K_V_1.2–K_V_1.5. However, both isoforms were suggested to provide new hints for understanding the biological mechanism of cardioprotection [102]. Recently, the affinity of RIIIJ to its K_V_1 channel target was investigated and disclosed a selectivity on two functional K_V_1 complexes in mouse DRG neurons. Similarly to K-CnTX, Conk-S1 and Conk-S2, which are able to discriminate different K_V_1, they were suggested to have therapeutic potentials for the treatment of hyperglycemic disorders. In fact, they were specifically effective in enhancing insulin secretion and lowering glucose levels due to the target of K_V_1.7 channel. It was demonstrated that Conk-S1 treatments may modulate pancreatic β-cell excitability at high glucose concentrations, eliminating the risk of hypoglycemia. Hence, these compounds were candidates as therapeutic drugs for diabetes by lowering blood glucose levels [103,110].

K_V_ channels targeting is rarer in CnTX concerning the inhibitory activity exerted on both Na_V_ and Ca_V_ channels. For this reason, the I-superfamily has received peculiar attention, although no pathophysiological impact has been yet demonstrated. I-superfamily includes a class of peptides with four disulfide bridges that were isolated from venoms of eleven different Conus species. The functional characterization of these toxins showed their ability to block the K_V_ activity of subtype 1.1 and 1.3 channels and to inhibit or modify ion channels of nerve cells. Due to the structural organization of amino acid residues, it was suggested that they may exert different bioactivities and functions [104,105]. Nonetheless, a large number of studies were carried out on the bioactive CnTX superfamily structure; however, only a few indications have been provided on the physio-pathological impact of CnTX-targeting K_V_ channels and their potential therapeutic properties. In fact, some authors recently defined this lack of investigations as “zone of ignorance” in molecular neuroscience, highlighting the need for new in-depth studies on the advantages of using κ-CnTX as pharmacological tools in living cells [111].

At last, it is worth mentioning that ViTx from *Conus virgo* was the first CnTX member of a structurally new superfamily of conid peptides affecting vertebrate K^+^ channels. Electrophysiological voltage-clamp studies testing various ion channels indicated that this toxin was able to inhibit K_V_1.1 and K_V_1.3 subtypes. However, limited studies were performed on possible biological actions. Its impact and activity remains still unexplored, at present, compared with Na_V_ and Ca_V_ channels, and few therapeutic applications of CnTX modulated K_V_ channels have been identified [106].

Moreover, synthetic versions of conopeptides have been tested for therapeutic use. In the case of CGX-1051, the K_V_ channel inhibitor from *Conus purpurascens* evaluated for possible cardioprotective properties on mammalian models showed a clinically relevant dose-dependent reduction in infarct size in both rats and dogs [107].

## 4. Conclusions

It is known that millions of adults worldwide suffer from channelopathy-related pain sensation and pathologies. Therapies for lowering pain symptoms have serious expensive socio-economic impacts due to reduced work performances of people facing anti-pain therapies. Furthermore, current medications have been demonstrated to have reduced efficacy and safety and can even be toxic [112]. In addition, a warring association between neurological pains and other diseases, such as cancer-associated chemotherapy, HIV, and diabetes, has been shown. In addition, neurodegenerative disorders, such as Alzheimer’s, Parkinson, and multiple sclerosis syndromes, are often age-related diseases, which affect a large number of people around the world. These are known to be associated with aberrant neuronal excitability often related to ion channels malfunctioning. Therefore, an urgent need to discover and develop new effective pain treatments with almost no side effects has been recognized. In this line, a large field of studies has been focused on clarifying the mechanisms underlying the pathophysiology of pain syndromes. The use of CnTX as therapeutic medications is well known [113]; nevertheless, some limitations such as low bioavailability and vulnerability to degradation still impede their widespread clinical use. The use of CnTX as therapeutic medications and research tools is well known for the scientific and societal benefits provided by their clinical application. However, due to their proven toxicity in humans, in a recent review, a potential alarm for their misuse as biological weapons has been highlighted, describing in detail biosecurity concerns along with past and current regulations for CnTX use in medical and research fields [114]. Since, in the last decades, many cardiac, muscular, and neurological disorders have been associated with the malfunction of ion channels, the ability of many CnTX to target ion channel protein subtypes renders them promising pharmacological tools for non-opioid anti-pain therapy. CnTXs are isolated from a large amount of Conus venoms, and their related isoforms fascinate scientists and clinicians due to their enormous potential for diseases diagnosis and care. In this narrative review, we provided evidence of the modulation of CnTX on the three major and most well-studied voltage-gated ion channels. Interestingly, CnTXs targeting Na_V_, Ca_V_, and K_V_ channels exert different and, sometimes, opposite effects, generating beneficial effects that alleviate pain or inducing, especially in animal models, aberrant channel dysfunctions and damage in organ and system functioning. In fact, some CnTXs reported in this review, even if isolated from the same species, can exert cardio and neuroprotective effects other than analgesic and therapeutic actions, but in contrast, they may generate pathological diseases such as paralysis and death. What is noteworthy is that studies on animal models may be helpful for defining action mechanisms as well as binding and affinity among CnTX, ion channel function, and biological processes.

## Figures and Tables

**Table 1:** CnTX subfamilies targeting voltage-gated sodium (Na_V_) channel subtypes, functional impact, and pathophysiological activity.

Species	CnTX Subfamilies	Channel Subunit Targeted	Functional Impact	Pathophysiological Activity	References
*C. geographus*	μ-GIIIA	Na_V_1.4	block skeletal muscle channels	paralysis	[40]
*C. geographus*	μ-GIIIB μ-GIIIC	Na_V_1.1 Na_V_1.2 Na_V_1.4 Na_V_1.6	discriminate between muscle and neuronal channels	-	[41]
*C. bullatus* *C. catus* *C. consor* *C. magnus* *C. purpurascens* *C. stercusmuscarum* *C. striatus* *C.* *tulipa*	μ-CnIIIA μ-CnIIIB μ-CnIIIC μ-CIIIA, μ-MIIIA	Na_V_1	block channel conductance	paralysis (CIIIA)	[42] [43]
*C. purpurascens*	μ-PIIIA	Na_V_1.2 Na_V_1.4 Na_V_1.7	inhibit channel modulation	-	[44]
*C. stercusmuscarum*	μ-SmIIIA		irreversible block of NaV currents	nociceptive role	[45,46]
*C. striatus*	μ-SIIIA	Na_V_1.2	block of neuronal NaV current	analgesic activity	[47]
*C. tulipa* *C. kinoshitai* *C. striatus*	μ-TIIIA, μ-KIIIA, μ-KIIIB, μ-SIIIB	Na_V_1.1 Na_V_1.2 Na_V_1.3 Na_V_1.4 Na_V_1.6	affinity Na_V_ channels	analgesic activity	[48,49,50]
*C. marmoreus*	μO-MrVIA, μO MrVIB, μO MfVIA	Na_V_1.8	inhibit channel activity	analgesic activity	[51]
*C. radiatus*	ί-RXIA	Na_V_1.6	shift channel activation	-	[52]

**Table 2:** CnTX subfamilies targeting voltage-gated calcium (Ca_V_) channel subtypes, functional impact, and pathophysiological activity.

Species	CnTX Subfamilies	Channel Subunit Targeted	Functional Impact	Pathophysiological Activity	References
*C. pennaceus*	ω-PnVIA ω-PVIB	HVA Ca_V_	selectively but reversibly block HVA currents	-	[70]
*C. textile*	ω-TxVII	Ca_V_	block Ca_V_ currents	-	[71]
*C. geographus*	ω-GVIA	Ca_V_	irreversibly block Ca_V_ channels	-	[72]
*C. magnus*	ω-MVIIA ω-MVIIC	Ca_V_2.2 P/Q-type Ca_V_2.1 and Ca_V_2.2	inhibits channel activity blocks channel activity	analgesic on chronic pain neuroprotective effect	[73,74]
*C. moncuri*	ω-MoVIA ω-MoVIB	Ca_V_2.2	channel affinity	-	[75]
*C. striatus*	ω-SVIA ω-SVIB ω-SO-3	Ca_V_2.2 Ca_V_2.1 and Ca_V_2.2 N-type Ca_V_2.2	targeting binding affinity inhibition	paralytic effect lethal injection attenuates acute and chronic pain	[76,77]
*C. catus*	ω-CVIE ω-CVIF ω-CVID	Ca_V_ N-type Ca_V_2.2	affinity antagonist activity	inhibition of nociceptive pain; reducing allodynic behaviour alleviates chronic neuropathic pain reduce allodynic behaviour	[78,79,80]
*C. fulmen*	ω-FVIA	N-type Ca_V_2.2	inhibition	reduces nociceptive behaviour, neuropathic pain, mechanical and thermal allodynia	[81]
*C. textile*	ω-CNVIIA	N-type Ca_V_2.2	inhibition	blocks neuromuscular junction, paralysis, death	[82]
*C. pergrandis*	α-PeIA	GABAB receptors coupled to N-type Ca_V_	blocking activity	analgesic activity	[83]
*C. victoriae* *C. regius*	α-Vc1.1 α-RgIA α-AuIB α-MII	GABAB receptors coupled to N-type Ca_V_2.2.	inhibition	analgesic activity on sciatic nerve ligation injury; allodynia relieves	[84,85,86,87]

**Table 3:** CnTX subfamilies targeting voltage-gated potassium (K_V_) channel subtypes, functional impact, and pathophysiological activity.

Species	CnTX Subfamilies	Channel Subunit Targeted	Functional Impact	Pathophysiological Activity	References
*C. striatus*	kA-SIVA	K_V_	block	spastic paralytic symptoms	[98]
*C. purpurascens*	K-PVIIA	K_V_1.3	inhibition	therapeutics for multiple sclerosis, rheumatoid arthritis, diabetes, and dermatitis	[99,100]
*C. radiatus*	kM-RIIIK K-CnTX RIIIJ	Human K_V_1.2 K_V_1.2–K_V_1.5	block target	cardio-protective action no activity	[101,102]
*C. striatus*	K-Conk-S1; K-Conk-S2	K_V_1.7	target	therapeutics for diabetes	[103]
*C. capitaneus* *C. miles* *C. vexillum* *C. striatus* *C.* *imperialis*	I-superfamily conus peptides	K_V_1.1 K_V_1.3	block	-	[104,105]
*C. virgo*	ViTx	K_V_1.1 K_V_1.3	inhibition	-	[106]
*C. purpurescens*	CGX-1051	K_V_	inhibition	cardioprotective	[107]

## Data Availability

Not applicable.
